# Topics of Public Interest

**Published:** 1917-12

**Authors:** 


### TOPICS OF PUBLIC INTEREST

**Centenarians.** Mrs. Annici Hawyer, born at Grafton, Vt., celebrated her 104th birthday at Aledo, Ill., Oct. 20. She was married twice, had five children, all dead, and is in good health. Mrs. Jenette Lincoln of Attica was 102 years old, Oct. 20. Mrs. Sarah Libby of N. Y. City was 100 years old Nov. 6.

**German Tonnage** of vessels was 3,153,724 in 1913. Of this 2,655,496 was steam.

**Baseball.** Several hundred thousand are said to have tried to see one of the final games between Chicago and N. Y. Nationals. 27,323 were admitted, paying $69,403—a good index of prosperity. It is just possible that the habit of showing enthusiasm for vicarious athletics and the popular approval of that enthusiasm, may account for the vicarious attitude which many take with regard to military service.

---

**Flour Consumption and Prices in England.** The antebellum consumption of wheat flour was 238 pounds per capita, the flour being 71% of the gross wheat, the remainder being used for feed, etc. On Sept. 1, 1917, 81% extraction and the use of 20% of other grains in the flour was made compulsory, with the expectation that the per capita consumption of wheat flour would be reduced to 119 pounds a year. The price of flour is standardized at $7.38 a barrel, about 2/3 of the regulated price in the U. S. Bread is fixed at 5 cents a pound loaf and 18 cents a 4-pound loaf. A very practical question arises for our country: We produce considerably more than our domestic consumption and still pay 150% of the English price for flour and 150-200% for bread. Even in peace, we paid nearly 80% of the war price for flour in England and 125-150% of the war price for bread. Of course, the war flour and bread are not pure wheat but this makes no practical difference in dietetic value and, so far as the ultimate consumer is concerned, other cereals, however much cheaper and abundant wholesale, usually have been and still are more expensive than wheat. We cannot make these facts seem right. P. S. This is a good illustration of the way news is allowed to filter through to the people. After letting them wonder and perhaps grow discontented with our own government, the explanation follows in two or three weeks that the English government pays a subsidy and that, otherwise, flour would be almost as expensive in England as in the U. S.

---

**Alvarenga Prize.** The award for 1917 has been made to Dr. Wilburt C. Davison of Baltimore for his essay "The Superiority of Inoculations with Mixed Triple Vaccine (B. typhosus and B. paratyphosus A & B) over Successive Inoculations of the Single Vaccines, as shown by Agglutination Curves in Men and Rabbits." The next award ($250) will be made July 14, 1918. Essays in competition must be typewritten in English, identified by a motto with sealed name and address, and submitted to the College of Physicians of Philadelphia before May 1.

---

**Biopsy,** rendered available in suspected cancer, by the Cancer Commission of Harvard University and by the N. Y. City Health Dept. is condemned by the Medical Record, Oct. 27. Without being able to speak with authority on the matter, we have understood that the general consensus of surgical opinion has long been to the effect that diagnostic section of a suspected malignant growth should be made only when the patient is prepared for immediate operation is positive results are obtained from the examination which should, of course, depend upon technic rendering prompt microscopic inspection available. We would welcome an expression of opinion on this subject which is reopened in a very practical way by the action of the laboratories referred to.

---

**Average Professional Clienteles.** Dr. A. J. Orenstein, of Johannesburg, South Africa, a Jefferson graduate, contributes a very interesting article on Medical Education in the U. S. to the Med. Jour. of C. A., June, illustrated with diagrams. Incidentally, he states the ratio of physicians to population in various countries as follows: America (assumed to mean U. S. and Canada together) 1: 691; Eng. 1: 1,000; Germany 1: 1,940; Austria 1: 2,120; France 1: 2,834. Of course, these are ante-bellum statistics but they should assuage the anxiety regarding depletion of the American profession, expressed by so many persons without due consideration of economic facts.

---

**Army Medical Service Statistics.** The Surgeon-General's Office kindly sends the following statistics to Oct. 23. We understand that the Dental Corps is practically complete and that no more commissions of any kind will be issued from the Adjutant-General's Office till about the middle of November. Active Duty: Medical Regular Corps, 820; Sanitary Corps, 375; Ambulance Service, 46; Reserve Corps, 12,357; National Guard, 1,516. Total, 15,174.

Medical Reserve Corps: Total recommendations 17,645; declinations recorded 1,233; discharges 218; acceptances, 535 majors, 2,291 captains, 11, 411 lieutenants, total acceptances 14,237 (leaving nearly 2,000 recommendations not acted upon and nearly 2,000 commissioned but not yet assigned to duty).

Dental Reserve Corps: Total recommendations 4,696; declinations 32; discharges 3; acceptances 3,919. (The comparison is, unfortunately, to the detriment of the medical profession).

National Army, Sanitary Corps: Total recommendations 427; declinations 10; acceptances 382. Ambulance Service: Total recommendations 91; acceptances 49.

The Regular Army Medical Corps (in addition to 1 major general and 4 brigadier generals) now consists of 63 colonels, 215 majors, captains, and 131 lieutenants. It includes a dental department of 178 and a veterinary of 118.

The National Guard, beside the 1,516 medical officers, has 320 dental and 85 veterinary surgeons.

---

**Post Office Rulings.** We are asked to call attention to the increase in rates, 2 cents for postal cards, 3 cents for letters. Rates in the same city remain unchanged. Letters to the American Expeditionary Force, by name, rank and military organization, as for domestic mail. Limits of weight of parcels, 7 pounds. Do not abbreviate "American" as there is an Australian Expeditionary Force also. The BUFFALO MEDICAL JOURNAL can be remailed at 1 cent, either in this country or to any one in the American Expeditionary Force. Ordinary literary magazines (but not medical) can be put to good use without specification of addressee, by affixing a 1 cent stamp and handing to any postal employee. Such magazines will be sent to camps, in this country or abroad and distributed to soldiers' reading rooms.

---

**Guide for Formulating a Milk Ordinance.** Those interested should apply to the U. S. Dept. of Agriculture, at Washington, for bulletin No. 585.

---

**Automobile Accidents** for the first nine months of 1917 have caused more deaths than typhoid. The latter had an average of 3,934 cases and 590 deaths for the average corresponding period of the last five years but declined to 2,840 cases and 434 deaths in 1917. Automobiles caused 755 deaths, 155 in September alone.

---

**Notice for Reserve Officers.** The Surgeon-General advises physicians commissioned in the Medical Reserve to continue their practices, as assignments to active duty may be delayed. At least 15 days' notice will be given.

---

**German Losses.** Ledebour, the Independent Socialist, announced in the Reichstag, late in October, that the German deaths for the first three years of the war were 1½ million, wounded 4 million, of whom ½ million were permanently crippled, absolutely invalided 2 million. Prisoners and missing, not included but conceded by German military reports: ½ million (a low estimate). This totals 4½ million definitive losses, although Ledebour stated them at 6 million without inclusion of prisoners. The additions from the 1915-17 classes would be about 1½ million, leaving the net definitive losses about 3 million, at a conservative estimate. It is, however, a fair estimate that the initial militia of any country is 10% of the population (about 6½ million for Germany) and that the uprising generation will just about compensate for deaths in the army from non-military causes, normal degrees of invalidism, etc. It would not compensate for the formerly prevalent camp diseases but these have been eliminated on both sides, except for the Balkan states and possibly Russia. Theoretically, the army would increase, if there were no military casualties at the same rate as the total population but in no ordinary country could the increase of births over deaths, carried forward year by year as increase in the population of one age over that of the next higher year, be more than 1% and practically, this increase would be more than offset by the increased invalidism of the higher ages in service. Thus, as a general principle, a country will be exhausted in the military sense when its aggregate definitive losses in a war of reasonable duration, equals its initial militia. Of course, there are numerous other factors to be considered in estimating the duration of the war, economic, political, psychologic, etc., as well as the purely military possibilities of the prior discovery of superior destructive means by either side. Toward the close of a war by attrition such as the present, there must be considered as disturbing factors; on the one hand, a drawing upon capital or unaccrued interest in the form of extending the age limits of service; on the other hand, a decisive victory or defeat such as was characteristic of former wars, without reference to man power in an exact quantitative sense. It is obvious that, in calculating attrition, only the side of smaller numbers need be considered, unless the attrition occurs at decidedly different actual and proportionate rates on the two sides. The calculation may further be reduced to Germany both as the dominant power in the Central Allies and because each of her allies, even including possible additions, is or would be engaged to the extent of the respective man power. It may be assumed that the final contest, so far as Germany is concerned is on the western front and it requires little geographic and political knowledge to see that the minimum defensive line—barring a final backing into a corner or forming a ring—is about 300 miles, requiring from 1½ down to 1 million effective troops. Thus, on an attritional basis, the duration of the war may be estimated at about 3 years from Aug. 1, 1917. An estimate on any other basis requires the gift of prophecy.

---

**Almost Literally a World War.** Of the total estimated population of the globe, 1,691 (millions understood in this and following numbers), the strictly neutral countries have a population of 132, those which have broken relations with but have not declared war on the central powers, 40. Central Allies, 177. Entente Allies 1,342. Excluding German colonies but including Asiatic Turkey, the Central Allies have a population of 143. It is extremely difficult to estimate the population of the Entente allies which can actually be drawn upon but, excluding Japan, Siam, Brazil and others which have not yet actually engaged in hostilities except by seizure of ships, etc., in their own territory, excluding Siberia and certain colonial possessions of Great Britain, France, Italy and the U. S. with sparse white populations and unavailable native populations (like the Philippines) the potential Entente population is somewhere between 500 and 700.

Very approximately, Austria-Hungary (50), Bulgaria (5) and Turkey in Europe (2) may be balanced against Italy (36), Greece (5), Serbia (4) and Roumania (7), with some allowance for Russia (117 in Europe). Asiatic Turkey (19) may be balanced against the Asiatic, African and Australasian parts of the British Empire (400-500 but only a small percentage essentially British). This leaves Germany (67) for the western front against Great Britain (46), Canada (7), France (46), Belgium (7), Portugal (6), U. S. (100), total 212. It is not meant that forces will actually be opposed to each other on this geographic basis but merely to show potential population strengths, divided roughly according to actual war fronts.

The total land area of the world (including rivers, lakes, etc., but excluding the oceans and principal seas is estimated at 57 million square miles. From this amount, about 7 is practically excluded as non-inhabitable polar regions, 5 as deserts, leaving the gross inhabitable area of the world 45. Of this, the Entente allies have about 27, the Central allies about 5.

---

**Health Insurance.** The Am. Assn. for Labor Legislation asks us to note that the British Medical Profession approves health insurance. The fact is that the British Medical Journal, commenting on the statement of F. L. Hoffman of the Prudential Ins. Co. that health insurance has been a failure in England, makes this guarded statement: "…. that insurance in England is a failure is certainly open to dispute. Even if it were admitted that the results have not reached expectations, it might still be argued that any such comparative failure arose from the insurance not going far enough, or from details in administration." The objection to health insurance is not on general principles but on account of omission of provisions for protecting medical labor; in other words of exploiting one form of labor for the benefit of another. Complaints of overwork and underpay from the British and German medical professions have been numerous and are apparently well grounded. The British Medical Profession, so far as it represents those especially interested, does not approve health insurance as it is administered. The American profession is divided on the matter but pretty well united on the contention that insurance should deal justly with all forms of labor, as well as with other interested parties, that it should not apply to the well-to-do simply because their work is classified as labor, that the medical profession should be adequately represented on all boards of control and that the work should be adequately compensated.

---

**Mayo Foundation Experiments on Animals.** A press story has been current that the Health Officer of St. Paul has refused to surrender dogs from the city pound to this institution on the ground that they must be humanely killed and the inference has been drawn, in some cases that the Health Officer, Dr. Justus Ohage, was an antivivisectionist, in others that the experiments were of a cruel nature. An inquiry directed to Dr. Ohage shows the exact status of the question: The city Charter of St. Paul provides explicitly that unmuzzled and unlicensed dogs must be killed promptly and by the Pound Master.

---

**The Moratorium.** B. R. Denny Chairman of the Physicians' Lease Committee of the Chicago Rotary Club announces that the wide publicity given the subject in medical journals has aided the successful prosecution of the work. Senator Chamberlin has introduced a bill, No. 2859, providing for a moratorium in the case of soldiers and sailors, and applying to leases, mortgages and life insurance. A moratorium is a two-edged sword and should be restricted so as to be just to both parties concerned. The medical profession well knows that there are numerous dead beats, disposed to take advantage of any technicality to defraud a creditor. Reasonable delay in the matter of rents, interest, premiums, etc., and prevention of sudden eviction, foreclosure and removal of insurance protection should, of course, be given those in military service. The indefinite free occupancy of premises as tenants or nominal owners may jeopardize the rights of others and diminish the potential taxing power of cities and states or else lead to ultimate confiscation of property. In many instances, this damage will be done to men themselves in military service. The only fair way to deal with the matter is to rebate taxes to the extent of actual losses by the moratorium on rents and interest, thus charging a legitimate public loss for military purposes to the community instead of to those who happen to have invested in certain forms of property. Unpaid premiums should of course, be deducted from insurance. Any moratorium law should also be executed with due regard to actual conditions so as to avoid suffering but not abet à dishonest person, even if patriotic.

---

**The Hahnemann Hospital of Rochester** opened Diagnostic and Research Laboratories, including general pathology and bacteriology, chemistry, Roentgenology and dental research, Nov. 1. A complete hospital unit for the study and treatment of diseases of metabolism is, at the same time, established, under the direction of Dr. John R. Williams. This consists of a group of private rooms and private wards, a diet kitchen and laboratories. Clinics on kidney disease will be held Tuesdays, and on diabetes on Fridays, 3-4 p. m.

---

**Meeting the Demand for Medical Officers.** The Regular Army and the National Guard have about 300,000 enlisted men each, requiring, according to regulations, 7 medical officers per 1000, total 4,200. The former has about 800, the latter about 1200, balance required 2,200. The first draft of the National Army—500,000—will require 3500 more medical officers. Total required from Medical Reserve Corps: 5,700. Total available (commissions accepted) Sept. 17: 11,156.

It should be considered, however, that the full demands of the army are 10 medical officers per 1,000, to include those at base hospitals—3,300 more. This requirement is largely met by the Red Cross. It will be seen that the volunteer system secured, shortly in advance of the assembling of the first draft, just about enough physicians to supplement the needs of the Regular Army, and the National Guard, to provide for the first two draft contingents and to leave enough over to allow a liberal margin for supplementing the Red Cross at base hospitals. This speaks well for the profession but the volunteering should continue, to provide an adequate reserve for the future.

There are two other important factors to consider: It is more than likely that the U. S. will have to provide medical men for the Allies and a liberal allowance must be made for replenishing the quotas mentioned. This last statement does not mean that a large percentage of medical officers will be killed or permanently disabled by war but the normal death rate will be close to 2% per annum, without any military risk at all; the radical change of duties and mode of living will undoubtedly reveal a far larger proportion of those unfit for military service for physical reasons, incapacity for discipline, etc., than among men selected a few at a time and trained for many years for the regular service. It is probably a conservative estimate that 5% per annum will be required simply to keep the numbers full for the above mentioned branches of the army, even if we disregard entirely the question of strictly military risk.

There is one more point that should be emphasized—and we emphasize it especially because we have presented some simple arithmetical calculations which might be interpreted to mean that the medical profession has done its entire duty and can now relax its efforts. The Surgeon General, unquestionably with the approval of other representatives of the army, including the chief executive of the nation, has repeatedly announced the need of a Medical Reserve Corps of at least 20,000. The medical profession should not stop short of this number. It should regard the preceding arithmetical calculations as of purely academic interest, satisfying for the time being only. It should not question whether the apparent discrepancy means an excessive war risk, or whether the ultimate intention is a far greater army than has yet been announced, or whether the needs of our Allies are greater than we imagine, or whether a wide margin of safety is all that is wanted. The medical profession is composed of a class of men who should lead in the comprehension of what war means and who should realize, not only in the intellectual sense but in the literal, practical sense that the best way to preserve liberty in a free country is to obey the proper authorities, promptly and without question, in the present emergency. We do not oppose the draft system for the medical profession but we trust that the actual necessity of a draft as a means of securing the desired number of medical officers will be anticipated by an ample number of volunteers.

---

**"Command" in the Medical Dept. of the Army.** Some months ago, we called attention to the fact that the medical department of the army was very far from being a bureau and that it was second in numbers only to the quartermaster's department. It is now, or will be as soon as the enlistments automatically provided are completed, the largest staff department in the army. With the Regular Army, National Guard and first draft of the National Army mobilized (Total about 1,100,000) the Surgeon-General will exercise whatever is the proper technical substitute for "command" over 11,000 commissioned medical officers, 1,100 dental officers, not to mention a considerable number of special appointees, 55,000 enlisted men and a considerable number of female nurses. The Surgeon-General, as a well merited recognition of personal services, at present ranks as major general but the office itself carries the rank of a brigadier general. A major general of the line (according to the latest available statement) exercises command over a division of about 27,000 men, including somewhere in the neighborhood of 1,000 officers. There certainly is need of revising the matter of rank for the medical department in accordance with actual facts.

---

**Old Age and Dependency.** A statement has been going the rounds of various journals, that of 100 healthy men at 25, 36 will be dead at 65 and of the remaining 64, 1 will be rich, 4 wealthy, 5 supporting themselves by work and 54 dependent on private or public charity. According to the U. S. Life tables, about 47 will be dead and 53 will be alive at 65 and these numbers are changed by only about 1, if we restrict the term healthy to those alive and 25 who survive to the age of 30. The economic status of the elderly is too important sociologically to be rated according to statistics which are, at the outset, so inaccurate. We know of no statistics that are reliable upon which the economic condition of elderly persons can be based but we are very certain that half of the non-dependent men of 65 are neither very rich nor wealthy. On the other hand, we are equally certain that there are not over 5 dependent men of 65 for every one who is earning a living or living on his own savings. Now that the subject has been broached, will not someone furnish an accurate statement of facts?

---

**The Official Bulletin** (the news (?) paper published by the U. S. Government) estimates that there are 1200 animals at each cantonment, producing 120 tons of manure a day. Either the animals have polychezia or there are a good many more animals at a cantonment, the latter being more probable as the animals for an infantry regiment on the new basis of 3,755 officers and men, number 399 according to another issue of the same Bulletin. At any rate, the sales of manure for 11 cantonments are said to be at the rate of $240,000 a year. Experiments at Chillicothe show that the garbage from 10-15 men will support one pig and it is estimated that 13 cantonments will yield nearly 19,000,000 pounds of pork which is worth about double what has been obtained from the direct sale of garbage, at a low price per pound. Garbage incineration, on the other hand would cost about $700,000 for installation and about $600,000 per year for operation.

**Increase of Male Births in England and Wales.** For the year July 1, 1914-June 30, 1915, ratio of boys to girls, 1040: 1000. Quarter ending with March 1915, 1032:1000; quarter ending with Dec. 1915, 1044:1000; quarter ending with March 1916, 1050:1000; June, 1051:1000; Sept 1045:1000; Dec. 1916, 1050:1000. Year July 1, 1915-June 30, 1916, 1047: 1000. This is the highest ratio of boys to girls recorded. We understand that German statistics have shown an even greater increase of male births so that the old notion that war tended to compensate for losses in the male population, by the preponderance of male births, has received considerable support.

---

**Liberty.** Captain Max V. Thierichsen, formerly commander of the S. S. Prinz Eitel Friedrich has been convicted of violation of the Mann law, acquitted of smuggling for taking several chronometers from the ship to land, also charged with sending improper matter through the mail, to another woman. He is probably convinced of our insincerity in waging a war for liberty. Whether the first offense was literally white slavery or voluntary immorality, does not appear. We disapproved the Mann act, as far as its general interpretation is concerned, at the outset, not because we are in league with vice but because of the obvious tendency to develop blackmailing, as has since been shown to have actually occurred, and because we feel that great reluctance should be shown by legislators to curtail personal liberty except when there is a very direct involvement of the rights and safety of others. Since the law has been construed as going far from the regulation of White Slavery, it is pertinent to inquire why it has been interpreted as applying only to one sex in regard to immorality, especially as its application to both would automatically eliminate much of the trouble with blackmail. There is a far broader significance to the general conception of Liberty: In any civilized community, especially if a fair degree of density of population exists, the ordinary doings of individuals must be regulated, for mutual safety, convenience and efficiency, in about the same ways and to about the same degree, whatever the form of government.

---

**Civil Service Positions.** Almost every month, we receive notices of state and national vacancies, too late so that they can be published in time for examination. Many of these are highly desirable and carry adequate salaries. Others are especially available for men desiring positions for research or experience and afford at least temporary support. Generally speaking these positions are numerous and more desirable than formerly. We would advise anyone who is on the look-out for such a position to telephone us so that we can give personal notice when the announcements are received too late for publication.

---

**Expenses of Medical Officers.** The statement is still going the rounds of the medical press that physicians entering the Medical Reserve can live on $25 a month and send the rest of their salary to their families. The facts are about as follows: Mess expenses at training camps vary from $5 to $10 a week. Some messes have gone to considerable extra expense for tableware. It is altogether likely that, for service abroad, food will be found nearly or quite as expensive under war conditions as in this country, possibly more so. There will be the temptation, either to relieve monotony or to keep up with the crowd, to indulge in considerable extravagance for meals, wherever the officer is located. The entire necessary outfit of clothing, aside from underwear which it is presumed the medical officer already has, but including shoes, rubber boots, slicker, overcoat, cotton and wool uniform, flannel shirts and insignia can be got through the quartermaster and reasonable dealers for a total of about $60. The same outfit, secured from fancy tailors and dealers in military supplies, and selecting high-priced materials, may very easily cost ten times as much. A judicious selection of certain goods from the quartermaster and recourse to good but moderate priced tailors, according as the garments are intended for rough use or dress, will give a very satisfactory equipment for $150, possibly $100. The necessary expense for blankets, cot, etc., need not exceed $15, and it is doubtful whether medical reserve officers will be required to have these items until they are ordered to camps or to actual service where they can be obtained from the quartermaster. The long list of items more or less required, such as watch, compass, knife, fountain pen, memorandum books as well as trunks and suit cases, stationery, etc., are such as most men already possess. Replacement of equipment will depend largely on unforeseen conditions and the personal equation. We understand that one of our national guard officers (not in the medical corps) has bought five expensive uniforms this summer and, on the other hand, others have apparently not had to replace their uniforms at all. As nearly as we can solve the problem, the personal expenses of the reserve officer when called into more or less active service, will be about what they are at home, less such obviously dispensable things as automobiles, with recreation and food plus or minus according to relative degrees of indulgence at home and in camp or the field. But it is absurd to tell him that he can live on $25 a month and he will have to economize closely to live on $50 a month.

---

**Military Discipline and the Medical Reserve Officer.** Some civilian physicians have expressed the fear that military discipline will prove disastrous to their personal comfort and even render service impossible by subjecting them to punishment or actual discharge for errors innocently perpetrated. From regular army officers, members of the national guard who have seen service in the Spanish war or recently on the border and various other sources, it appears that there is a general disposition to overlook technicalities in dealing with inexperienced men and that no civilian of good sense need fear on this score. The medical reserve officer will be mainly under the supervision of his professional confreres and ultimately the Surgeon-General. Recently, we heard a little anecdote that has a good deal of significance. A young physician who served on the Isthmus under (then) Colonel Gorgas said that when he had been on duty a week and was realizing his unfamiliarity with the work and his general lack of importance and wondering whether he could get along in the service, he met and saluted the Colonel and was pleasantly surprised not only at the courteous recognition of his salute but at being called by name. Toward the completion of his service, the same superior showed the same personal interest by notifying him of an opportunity to return to the states as ship surgeon to San Francisco. Military discipline is necessarily strict and requires some readjustment of civilian habits of intercourse but the reserve officer may feel confident that he is in the hands of men who will not unnecessarily and arbitrarily emphasize this point but will look after his best interests so far as possible.

---

**Automobile Upkeep in Europe.** The principal practical objection to life in the U. S. is the enormous prices in comparison with normal European standards. Even things that are inevitably higher wholesale are usually considerably cheaper retail. Under war conditions, the following prices are said to be current: Tires, $90 in Great Britain and France, $100 in Russia and Italy, $125 in Spain, $350 in Holland, $320 in Denmark, $460 in Norway, $550 in Sweden. It is said that tires are absolutely unobtainable in Germany—except rope substitutes and probably a few for the Emperor. Gasoline is 95 cents a gallon in Great Britain, ranges from $1 to $1.75 in the allied and neutral countries, and is held at $6 in Austria and Germany.

**Chamberlain Bill for Universal Military Training.** The Clinical Congress of Surgeons and State Committees of the Medical Section, Council of National Defense, at meetings in Chicago late in October, adopted resolutions favoring this bill . . At least six months of intensive military training of all young men in their nineteenth year, to become operative as soon as the army cantonments are available, also recommending physical training in schools.

---

**Niagara Falls Memorial Hospital.** The Louise Section was opened November 11, two large wings having been added at a cost of eighteen thousand.

---

**Relative Value of Flour and Potatoes.** We estimate on a caloric basis that an economic balance is struck when the price of an eighth barrel sack of flour is the same as of a bushel of potatoes. This does not allow for the higher proteid content of flour.

---

**Pneumonia.** The Metropolitan Life Insurance Co., reports 38,000 deaths among industrial policy holders in the last six years. It is claimed that lobar pneumonia causes more deaths than any other infectious disease and that it should be quarantined against.

---

**U. S. Medical Forces Attached to French and British.** November 8, there were 870 Medical Officers and 470 nurses serving with British and 40 ambulance sections of 46 officers and men each with French troops. 120 more ambulance sections will be formed and trained at Allentown, Pa., for service with the French Army.

---

**Railway Casualties.** 1916. The Interstate Commerce Commission reports 206,723 accidents including 10,001 deaths. 129,740 casualties including 525 deaths were not directly due to trains. Of the total casualties 176,923 were employees; 8,008 passengers; 11,791 other persons including trespassers. The deaths were respectively 2,941; 291; and 6769.

---

**War Losses.** Early in the war, we expressed the opinion that the deaths were being exaggerated, that anything approaching male depopulation of any country was not to be expected, and that the prevalent statements as to the excessive proportion of fatal wounds, based on the use of high explosives and massive projectiles and the relatively greater exposure of the head in trench warfare, should be seriously questioned. The most accurate statistics for the first three years of the war just about equaled the killing on paper, published May 31, 1915. Military authorities now agree in placing the ratio of killed (including delayed fatal results of wounds) to total casualties at 20%, the standard rate for most modern wars. The French fatalities have ranged from 5.41% of the total mobilized French forces for the "period" of the battles of Charleroi and the Marne, down to about 1.1% per annum or to 1.28% for total losses in killed, wounded and prisoners for the last six months of 1916. Even early in the war, the results of wounds were highly favorable: for the French army, early in the war, 54.5% returned to duty within a short time, 24.5% returned to duty after convalescence at home, 17% still under treatment with expectation of complete recovery, 1.5% recovered unfit for further duty, 2.5% died. Still better results are said to have been achieved later and the German reports are equally favorable. These wonderful results as compared, for instance, with those of the Civil War, are due mainly to the practical elimination of tetanus, septicaemia and intercurrent infections, as typhoid, and the restriction of amputations to the severest mutilations. While alarming and probably exaggerated reports of prevalence of tuberculosis and syphilis are prevalent, the ordinary camp diseases have been almost entirely eliminated and the statement has been made, especially for the British troops, that deaths from sickness are less than in civil life for a comparable young male population. The killed and fatally wounded of the Civil War represented an annual death rate of almost exactly the same percentage as given above (1.1%). The Civil War military casualties were really far greater, proportionately to the troops actually under arms at any given period, and still greater on the basis of actual combatancy, although it is practically impossible to reduce this statement to accurate figures on account of the vastly different conditions, especially as to continuous fighting. The deaths from disease for the Civil War were almost double the military fatalities and, including accidental deaths and those of prisoners, the direct military fatalities were less than a third of the total deaths. The total losses, killed, died, wounded, prisoners, for the Civil War amounted to about 8% per annum, based on the ultimate total number of troops raised. Those for France amount to about 2.56% per annum, based on the troops mobilized at present.

These statements are made with the qualification that the present statistics may be incorrect or stated in such a way as to cause false inferences. But, so far as the premises are reliable, the conclusion seems to be warranted that the average risk of death from missiles and other means of attack, is about the same as for the Civil War, in spite of the prospect of more continued actual combatancy; that the chances of recovery from wounds is far greater, either as regards life or restoration to health; that the risk of death due to disease, etc., is practically the same as in civil life, perhaps less; and that the risk of any military unpleasantness, death from missiles or disease, wounding or capture, is about a third of that encountered during the Civil War.

---

**Meaning of the Word "Reserve."** We understand that some reserve officers have insisted on immediate service of a definite kind and, on the other hand, that others have insisted that they shall be kept permanently in reserve. The latter remind us of the man who proposed to a girl that she might add a tally to her list of rejections, but with the stipulation that there must be no question but that she would refuse him. A reserve of medical officers does not differ essentially from one of any other kind of military forces. It it equally worthless if it must be prematurely sent into action, or if it cannot be sent into action at all.

---

**Fort Porter,** Buffalo, has been selected by the Army as General Hospital No. 4, under the command of Major Thomas D. Woodson. It will be used at first as a training depot for base hospitals. At date of writing (Nov. 17) Base Hospital No. 23 in command of Major Guy V. Rukke is waiting travel orders. Base Hospital No. 10 of New York City and another are also mobilized here for the present.

